# An effective method of large scale ontology matching

**DOI:** 10.1186/2041-1480-5-44

**Published:** 2014-10-28

**Authors:** Gayo Diallo

**Affiliations:** University Bordeaux, ISPED, Centre INSERM U897, F-33000 Bordeaux, France

**Keywords:** Ontology matching, Life sciences ontologies, Entity similarity, Information retrieval, Machine learning, Semantic interoperability

## Abstract

**Background:**

We are currently facing a proliferation of heterogeneous biomedical data sources accessible through various knowledge-based applications. These data are annotated by increasingly extensive and widely disseminated knowledge organisation systems ranging from simple terminologies and structured vocabularies to formal ontologies. In order to solve the interoperability issue, which arises due to the heterogeneity of these ontologies, an alignment task is usually performed. However, while significant effort has been made to provide tools that automatically align small ontologies containing hundreds or thousands of entities, little attention has been paid to the matching of large sized ontologies in the life sciences domain.

**Results:**

We have designed and implemented ServOMap, an effective method for large scale ontology matching. It is a fast and efficient high precision system able to perform matching of input ontologies containing hundreds of thousands of entities. The system, which was included in the 2012 and 2013 editions of the Ontology Alignment Evaluation Initiative campaign, performed very well. It was ranked among the top systems for the large ontologies matching.

**Conclusions:**

We proposed an approach for large scale ontology matching relying on Information Retrieval (IR) techniques and the combination of lexical and machine learning contextual similarity computing for the generation of candidate mappings. It is particularly adapted to the life sciences domain as many of the ontologies in this domain benefit from synonym terms taken from the Unified Medical Language System and that can be used by our IR strategy. The ServOMap system we implemented is able to deal with hundreds of thousands entities with an efficient computation time.

## Introduction

With the wide adoption of Semantic Web technologies, the increasing availability of knowledge-based applications in the life sciences domain raises the issue of finding possible mappings between the underlying knowledge organisation systems (KOS). Indeed, various terminologies, structured vocabularies and ontologies are used to annotate data and the Linked Open Data Initiative is increasing this activity. The life sciences domain is very prolific in developing KOS ([1–4] are examples of such resources) and intensively using them for different purposes including documents classification [5] and coding systems to Electronic Health Records [6].

One of the key roles played by these KOS is providing support for data exchanges based on a common syntax and shared semantics. This particular issue makes them a central component within the Semantic Web, the emerging e-science and e-health infrastructure.

These KOS, which are independently developed at the discretion of various project members, are heterogeneous in nature, arising from the terminology used, the knowledge representation language, the level of semantics or the granularity of the encoded knowledge. Moreover, they are becoming more complex, large and multilingual. For instance, the Systematized Nomenclature of Medicine--Clinical Terms (SNOMED-CT) [7], a multiaxial, hierarchical classification system that is used by physicians and other healthcare providers to encode clinical health information, contains more than 300,000 regularly evolving concepts. Each concept is designated by synonymous terms, sometimes by several. Another example is the International Classification of Diseases (ICD), the World Health Organization’s standard diagnostic tool for epidemiology, health management and clinical purposes used to monitor the incidence and prevalence of diseases and other health issues. The current ICD-10 version contains more than 12,000 concepts designated with terms in 43 different languages including English, Spanish and French.

There is a clear need to establish mappings between these different KOS in order to make inter-operable systems that use them. For instance, the EU-ADR project [8] developed a computerised system that exploits data from eight European healthcare databases and electronic health records for the early detection of adverse drug reactions (ADR). As these databases use different medical terminologies (ICD-9, ICD-10, Read Codes, International Classification of Primary Care) to encode their data, mappings are needed to translate a query posed to the global system into queries understandable for the different data sources. Performing manual mappings between all the mentioned resources is not feasible within a reasonable time. Generally speaking, the data integration domain [9], the semantic browsing of information domains [10] and web services composition [11] are areas where the matching of knowledge resources is usually performed.

There is therefore a crucial need for tools which are able to perform fast and automated mapping computation between entities of different KOS and which can scale to large ontologies and mapping sets. Significant effort has been expended in the ontology alignment/matching domain. A matching system is defined by the Ontology Alignment Evaluation Initiative (OAEI) [12] as a software program capable of finding mappings between the vocabularies of a given set of input ontologies [13]. Formally, given two ontologies, a mapping is a 4-tuple [14]:


such that:

- *id* is an identifier for the given mapping;
- *e*_*1*_ and *e*_*2*_ are entities, i.e. classes and properties of the first and second ontology, respectively;
- *r* is a relation, e.g. equivalence (=), subsumption (⊒), disjointness (⊥) between *e*_*1*_ and *e*_*2*_.

Some metadata, including a confidence value, *w* (usually ⋲ [0, 1]), are often associated with the mapping.

In the following section we will briefly give an overview of different approaches and systems in line with the approach we propose in this paper. In particular, we will review approaches which use a space reduction strategy for large scale ontology matching and machine learning- (ML) based matching and briefly present systems evaluated recently for the largest task in the context of the international OAEI campaign. We will further discuss, in Discussion, systems for matching ontologies in the biomedical domain.

## Related work

Ontology matching is an active research area. Existing ontology matching systems use terminological, structural and semantic features for the computation of candidate mappings (please see [14–16] for a complete survey). Despite the advances achieved in matching relatively small size ontologies, the large scale matching problem still presents real challenges to tackle, due to the complexity of such a task. These challenges include efficiency issues in term of space and time consumption, the use of background knowledge, user involvement and the automated evaluation of the matching system [14, 17]. Therefore, approaches for ontology matching have been proposed in the literature including clustering and blocking strategies (reduction of search space), ML- based matching (in particular for reusing existing alignments or combing results for parallel matches), interactive alignment (taking into account the user) and the use of specialised background knowledge (in particular for the life sciences domain).

A structure-based clustering approach for the matching of large ontologies is introduced in [18]. The idea is to partition each input schema graph into a set of disjointed clusters before identifying similar clusters in the two schema graphs to be matched. The COMA++ system [19] is finally used to solve individual matching tasks and combine their results. Hamdi *et al*. provide TaxoMap [20], a tool which is based on the implementation of the partition-based matching algorithm proposed in [21] to find oriented alignment from two input ontologies. TaxoMap provides one-to-many mappings between single concepts and establishes three types of relationships: equivalence, subclass and semantically related relationships. The semantically related relationships denote an untyped link indicating the closeness of two concepts. Hu *et al*. [21] address the issue of aligning large ontologies by proposing a partition-based block approach for the matching of large class hierarchies. Their matching process is based on predefined anchors and uses structural affinities and linguistic similarities to partition small block input class hierarchies. In contrast to these divide-and-conquer methods, Wang *et al*. [22] use two kinds of reduction anchors to match large ontologies and reduce time complexity. In order to predict ignorable similarity calculations, positive reduction anchors use the concept hierarchy while negative reduction anchors use locality of matching. A partial reference alignment strategy is used in [23] in order to partition ontologies to be aligned, computing similarities between terms and filter mapping suggestions. To test the approach, alignments provided by OAEI and from previous evaluation of the SAMBO system [24] are used.

On the other hand, Nezhadi *et al*. use an ML approach to combine similarity measures of different categories in order to align two given ontologies [25]. Their evaluation of different learning classifiers – K Nearest Neighbor, Support Vector Machine (SVM), Decision Tree (DT) and AdaBoost – on real life (small) ontologies for bibliographic references provided by the OAEI campaign [12], showed that using feature selection and a combination of AdaBoost and DT classifiers improves the F-measure. Ichise describes a framework which follows a SVM-based approach for ontology matching [26] while the GLUE system [27] applies a meta-learning approach in order to generate matching hypotheses using multiple local classifiers. These classifiers are trained first on different aspects of the models that are matched.

Some research works have addressed the user involvement issue in matching large ontologies. Lambrix and Kaliyaperumal [28] proposed an ontology alignment framework at large scale, which includes components from the SAMBO system [24], that allows a user to interrupt and resume the different stages of the ontology alignment task. Jiménez-Ruiz *et al*. [29] implemented in the LogMap system a strategy based on asking the user to interactively revise the candidate mappings arranged in a partial order based on their similarity.

The OAEI campaign has played an important role in the area of ontology matching. It is an international campaign for the systematic evaluation of ontology matching systems. Few systems, including GOMMA (Generic Ontology Matching and Mapping Management) [30] and LogMap [29], were able to complete, in the 2011 edition, the largest task of the campaign: the LargeBiomed track, which consisted of matching the Foundational Model of Anatomy (FMA) [31], the National Cancer Institute (NCI) Thesaurus [32] and the SNOMED-CT, with a good F-measure in a reasonable time. The (not) Yet Another Matcher or YAM++ system [33] joined these systems during the 2012 edition of the campaign. GOMMA [30] implements various techniques to match large ontologies in particular for the life sciences domain. It uses parallel matching on multiple computing nodes; composition techniques of previously computed ontology mappings; and finally a blocking strategy to reduce the search space. LogMap is a scalable ontology matching system which uses lexical and semantic indexing techniques and implements a reasoning-based diagnosis and inconsistency repair capabilities [29]. It further supports user interaction during the matching process. LogMap provides a lightweight variant called LogMapLt, which does not use reasoning nor repair facility and semantic indexing. YAM++, is a self-configuration, flexible and extensible ontology matching system which combines various techniques to perform mappings between two input ontologies [33]. The DT learning model is used to combine different terminological similarity measures, and a similarity propagation method is performed to discover mappings by exploiting structural information of entities. A semantic verification is used to refine computed mappings in order to eliminate those which are inconsistent. All these three systems obtained very good results for the task related to large ontologies matching during the 2012 edition of OAEI. Among the systems which were used at OAEI and are primarily dedicated to matching ontologies in the biomedical domain, the SAMBO system [24] achieved the best performance for alignment of the largest task (anatomy track) before the introduction of the LargeBiomed track. This system uses a terminological matcher (based on the textual descriptions of concepts and relations), a structural matcher based on the is-a and part-of hierarchies and domain knowledge based on the Metathesaurus of the Unified Medical Language System (UMLS) [34].

### Our contribution

We propose a generic approach to matching large ontologies. Our first contribution is an approach based on Information Retrieval (IR) techniques and an indexing strategy, in contrast to the previously presented blocking strategy, to address the challenge of scalability and efficiency of matching techniques. One of the novelties of the approach is the reduction of the search space through the use of an efficient searching strategy over the built indexes to be matched. The second contribution is the use of a new contextual ML-based strategy to provide candidate mappings to complement lexical (or terminological) candidate mapping generations. The third contribution is a fully implemented and evaluated system on standard benchmarks provided by the OAEI campaign. Eventually, general purpose background knowledge is used to improve the performance of the system and addresses the matching with background knowledge requirement [14]. In addition the current performance of ServOMap (described below) does not depend on any domain specific background knowledge.

The work presented in this paper is an extension introduced partly in [35] of the approach implemented within the ServOMap system [36, 37], a highly configurable large scale ontology matching system able to process large ontologies associated with multilingual terminologies. ServOMap takes as input ontologies described in standards languages RDF(S) [38], OWL [39], OBO [40] and SKOS [41] and provides equivalence mappings between their entities. It relies on an Ontology Repository (OR) system, ServO [42, 43], a system able to manage multiple KOS while providing indexing and retrieving features. It is based on the Lucene full text search engine API [44]. ServO provides an ontology management module for parsing and navigating ontologies and an ontology indexing and retrieval module, which implements the vectorial space model (VSM) [45]. Lucene is a highly and quickly scalable open-source library for IR. With the API, the data being indexed are stored as *documents*. A Lucene *document* represents a collection of fields. Thus, each *document* in an index contains one or more named fields. Each field corresponds to a piece of data that is either queried against or retrieved from the index during search.

Because it is based on the ServO system, ServOMap follows an IR-based technique [46] for computing of similarity between entities. An ontology is seen as a corpus of semantic *virtual documents* which represent the entities of the ontology. Specific fields are created to handle the different elements describing the entities of the ontology.

The rest of the paper is structured as follows. In Evaluation and results we give an overview of the method that we propose for the matching of large ontologies. In particular we detail the new contextual similarity computing strategy for the retrieval of candidate mappings based on the structure of the input ontologies. In Results we present the results obtained by our approach on an official dataset dedicated to evaluating ontology matching systems. We discuss the obtained results and the limitations of the approach then offer new perspectives on our work in Discussion before concluding.

From now on we use the generic term ontology (formally defined in the following section) to denote any KOS, ranging from simple thesauri to very formal ontologies.

## Methods

In this section, we describe in detail the overall process that is followed in our ontology matching approach. We start by introducing a formal definition of an ontology as used in the paper, based on the metamodel defined for ServO [42] and the primitives introduced in [47] adapted to the definition given by [48].

Then, we define the notion of descendant, ancestor and sibling concepts, and finally our notion of *virtual documents*.

**Definition 1 (ontology):** an ontology is a 5-tuple *O* = *<C*^*o*^*, R, H*_*r*_*, T, Lex >* and *R = R*_*I*_ ∪ *R*_*T*_ ∪ *R*_*D*_ such that:

- *C*^*o*^ is a set of concepts;
- *R*_*I*_ ⊂ *C*^*o*^ × *C*^*o*^ is a concepts taxonomy and *h* = (*c*_*1*_, *c*_*2*_) *∈R*_*I*_ means *c*_*1*_ is a subsumer of *c*_*2*_, the *is-a* relation;
- *R*_*T*_ ⊂ *C*^*o*^ × *C*^*o*^ × L_T_ is a set of transversal relationships where L_T_ is a set of relations labels;
- *R*_*D*_ ⊂ *C*^*o*^ × P × L_D_ is a set of attributes where P is a set of xml primitive data types and L_D_ is a set of relations labels;
- *H*_*r*_ ⊂ *R* × *R* is a taxonomy of relationships on *R*_*T*_ and *R*_*D*_;
- *T* is a set of (multilingual) strings terms that are concept labels (synonym terms);
- *Lex*: *T* → *C*^*o*^ is a function which associates concepts with their labels.

*R*_*T*_ and *R*_*D*_ are respectively object and datatype properties in the sense of web semantic languages. Some constraints can be associated with these properties, for instance the notion of functional property.

**Definition 2 (direct descendant concepts**, **):** given an ontology *O* and a concept *c*, the direct descendant concepts of *c* within *O* denoted  is the set:


**Definition 3 (direct ancestor concepts,****):** given an ontology *O* and a concept *c*, the direct ancestor concepts of *c* within *O* denoted  is the set:


**Definition 4 (sibling concepts,****):** given an ontology *O* and a concept *c*, the sibling concepts of *c* within *O* denoted  is the set:


We can now define the notion of *virtual document* that is subdivided into *direct virtual document* and *extended virtual document*. The notion of *virtual document*s from the RDF graph has been previously used in [49].

**Definition 5 (*direct virtual document*):** Given an ontology *O* and an entity *e ∈ O*, a *direct virtual document* or *dVD(e)* is constituted by the combination of its uniform resources identifier, the *uri*, obtained by *ID(e)*, the local name (*locName(e)*), the labels in different languages extracted by the inverse of the function *Lex* (*Lex*^*-1*^) and the set of annotations associated with it. Formally,
1

where *R*_*T*_(*e*), *R*_*D*_(*e*) give the properties attached to *e* and their information. *Dom(e)* and *Range(e)* give respectively the list of domains and ranges of the property *e* and *const(e)* gives the property constraints associated to *e* (e.g. functional property).

Now we define the notion of *extended virtual document* for a concept, which represents its virtual transitive closure.

**Definition 6 (*extended virtual document*):** given an ontology *O* and a concept *c ∈ O*, let’s assume that *Sup*_*Lex*_*(c)* = {*t* ∈ *T*| ∃ *c*_*i*_ ∈ *C*^*o*^, *c*_*i*_ *R*_*I*_ *c and t* ∈ *Lex*^- 1^(*c*_*i*_)} denotes the terms (labels) associated with the ancestors of *c* and *Sup*_*LocName*_*(c)*. = {*ln* | ∃ *c*_*i*_ ∈ *C*^*o*^, *c*_*i*_ *R*_*I*_ *c and ln* ∈ *locName*(*c*_*i*_)} denotes the local names of the super-concepts of *c* in *O*. The *extended virtual document eVD(c)* is constituted by *dVD(c), Sup*_*Lex*_*(c)* and *Sup*_*LocName*_*(c)*. Formally,
2

And for a property *p*, the *eVD(p)* is constituted by the *dVD(p)* and the local names of the super-properties of p*,* which belong to *H*_*r*_ in the sense of Definition 1*.*

**Figure 1:**
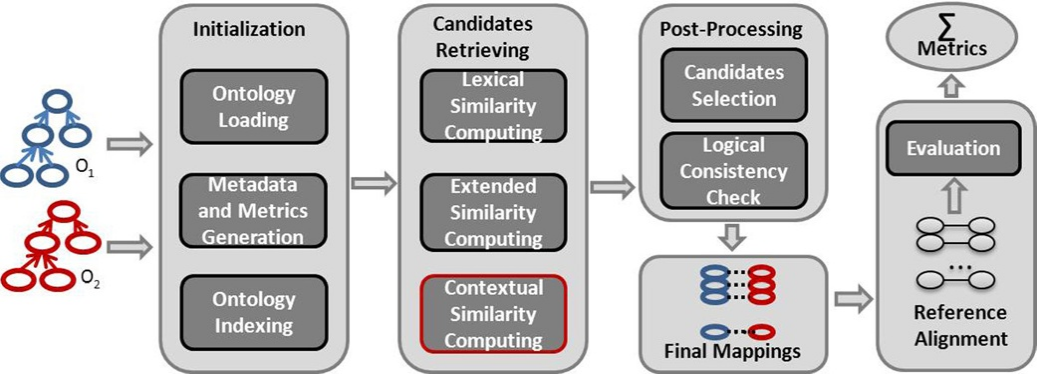
**Matching process of ServOMap.**

Let’s now detail the matching process. ServOMap relies on the use of IR techniques for ontology matching. In particular, it uses the VSM [45] on which the ServO OR is based. In the VSM, *documents* and queries are represented as weighted vectors in a multi-dimensional space, where each distinct index term is a dimension, and weights are *tf-idf* values. In the following sections, we detail the overall process of the approach as depicted in Figure 1.

### Initialisation phase

#### Ontology loading

The ontology loading step takes charge of processing the input ontologies. For each entity (concept, property), a *direct virtual document* from the set of annotations is generated for indexing purposes. We consider any ontology, regardless of its degree of formalism, as a corpus of *semantic documents* to process following Definition 1. Each entity (concepts, properties including both object properties and data type properties) is therefore a *document* to process. For each input ontology, we use ServO to dynamically generate a *direct virtual document* corresponding to any retrieved entity and instantiate the ServO metamodel. The objective is to gather the terminological description of each entity in order to build a vector of terms. Each *virtual document* has a set of fields for the storing its different elements.

The generation process is dynamic as each entity is described according to the features it holds. Thus, some concepts may have synonyms in several languages or may have comments whereas others may only have English terms. Some concepts may have declared properties (either object properties or datatype properties, etc.), therefore it may arise that some fields may not be instantiated.

#### Metadata and metrics generation

After the loading ontologies, a set of metrics are computed. They include the size of input ontologies in terms of concepts, properties and instances, as well as the list of languages denoting the annotations of entities (labels, comments), etc. Determining the input size helps in later adapting the matching strategy. We distinguish two categories regarding the size: matching two ontologies with less than 500 concepts each and matching ontologies with a number of concepts ≥ 500. Further, we pre-identify whether the matching problem is an entity level or instances level matching. The purpose of detecting the set of languages allows the use of the latter as the appropriate list of specific stopwords and the use of stemming during pre-processing.

#### Ontology indexing

**Figure 2:**
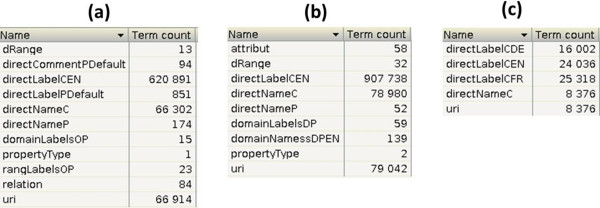
**Generated fields for respectively NCI Thesaurus (a), FMA (b) and TheSoz (c).**

As in the traditional IR domain, the purpose of the indexing step is to build an inverted index for each input ontology from the *virtual documents* previously generated. Each index contains a set of fields identified during the generation of *virtual documents*. Figure 2 gives an example of available fields for three different resources: a) the NCI Thesaurus; b) the FMA; and c) the Thesaurus for the Social Sciences (TheSoz) (c) which is used to index *documents* and research information in the social sciences (available at http://lod.gesis.org/thesoz/). This latter resource provides label terms in English (directLabelCEN), German (directLabelCDE) and French (directLabelCFR).

For each field, we can see the number of entries in this figure. For instance, there are 79,042 entries for the *uri* field of the FMA, which represents the number of entities of this ontology.

We proceed as follows to build the index. Each *dVD* is passed through a set of filters: stopwords removal, non-alphanumeric character removal (for harmonisation of the terms description), lowercasing and label stemming and converting numbers to characters. Indeed, we use a VSM -like system from the IR field to compare terms. Therefore non-alphanumeric symbols are removed in order to harmonise the description of terms. The conversion of numbers to characters contributes too in reducing mistakes during the matching process. For instance, for the two biomedical ontologies FMA and NCI, the Ninth_thoracic_vertebra of the FMA corresponds to the T9_Vertebra of the NCI thesaurus (knowing that the latter also has T8_Vertebra and so on).

In addition, to deal with the variation in naming concepts, labels denoting concepts are enriched by their permutation before stemming and after stopwords and non-alphanumeric character removal. We use permutation in order to deal with variation in naming concepts. Indeed, in the biomedical domain, many ontologies reuse the UMLS [34] to acquire synonymous terms. It is common to come across concepts denoted by all possible permutations between words of the terms. For instance, the concept “Myocardial Infarction” with the UMLS CUI C0027051 has among its labels: *myocardial infarction*, *heart attack*, *infarctions myocardial*, *attacks heart*, etc. The permutation operation is applied to the first four words of each label. We limit it to the first four words for two reasons: i) most terms used to denote entities that we encounter are of less than five words; ii) increasing this threshold affects the performance in terms of computation time without a significant gain in terms of F-measure. For instance, after enriching the term *thoracic vertebral foramen* we obtain, before stemming, the set {*thoracic vertebral foramen, thoracic foramen vertebral, vertebral thoracic foramen, foramen vertebral thoracic, foramen thoracic vertebral, vertebral foramen thoracic*}. The permutation process slightly increases the size of the index (around 20%). For instance, the index of SNOMED-CT is 22.8 Megabyte with permutation against 18 Megabyte. We can notice that the increase of the size of a Lucene index is not linear according to the input data due to internal compression strategy. In addition, our experiments show that the impact of the permutation on the precision is negligible compared to the gain in terms of F-measure. It is of the order of 2%. This low impact could be explained by the combined use of the concatenation.

Two strategies are used for indexing: *exact* and *relaxed* indexing. Exact indexing allows highly precise candidate retrieving. In this case, before the indexing process, all words for each label are concatenated by removing the spaces between them (e.g. *thoracic vertebral foramen* becomes *thoracicvertebralforamen*). In addition, for optimisation purposes, the possibility of indexing each concept’s *dVD* with information about the siblings, descendants and ancestors of the entity that it describes is provided.

Table 1 gives an example of three kinds of entries for an index: a concept, a datatype property and an object property. The example is taken from the *Thoracic_vertebral_foramen* concept and the *Outdated_meaning* datatype property of the FMA, and the NCI *Gene_Product_Chemical_Classification* object property. The permutations of the label of the concept are partially represented in the table to save space. The different entries have been passed through different filters according to the given field. As we can observe, for a datatype property we keep a *propertyType* field that indicates the constraint, if available (in the example, it is a functional property). For the concept, the local name (*directNameC*), the English label (*directLabelCEN*) and the *uri* are generated and indexed.

**Table 1:** **Example of the entries of the index for a concept, a datatype and object properties after pre-processing**

Entity	Field	Description	Value
Concept	directLabelCEN	Labels in English	thoracvertebrforamen foramenthoracvertebr foramenvertebrthorac vertebrforamenthorac
	directNameC	Local name	thoracicvertebralforamen
	uri	URI of the entity	http://bioontology.org/projects/ontologies/fma/fmaOwlDlComponent_2_0#Thoracic_vertebral_foramen
Datatype property	dRange	Range restriction	xsd string
	directNameP	Local name	outdatmean
	domainLabelsDP	Domains restriction (concept hierarchy)	concept name attribute entity
	propertyType	Constraint on the property	function
	uri	URI of the entity	http://bioontology.org/projects/ontologies/fma/fmaOwlDlComponent_2_0#Outdated_meaning
Object property	directNameP	Local name of the property	geneproductchemicclassif
	domainLabelsOP	Domains restriction	Gene Product Kind
	rangeLabelsOP	Ranges restriction	Gene Product Kind
	uri	URI of the entity	http://ncicb.nci.nih.gov/xml/owl/EVS/Thesaurus.owl#Gene_Product_Has_Chemical_Classification

### Candidate mappings retrieving phase

After the preliminary phase, which prepares the matching process, the main component for candidate retrieving is launched.

**Definition 7 (candidate mappings):** given two inputs, ontologies *O*_*1*_ and *O*_*2*_, their respective indexes, I_1_ and I_2_, and an optional background knowledge (denoted as *BK*), the candidate mappings, denoted as  is the union of the candidates generated using respectively lexical (denoted as  for concepts,  for properties), extended (denoted as  and contextual (denoted as ) similarity computing:
3

Knowing that the following property is satisfied: . Indeed, these different results are successive. In our strategy, each result provides candidates not previously found. Each set is constituted by a set of triples *< e*_*1*_*, e*_*2*_*, s >* such that  and  and *s* is the computed similarity between *e*_1_ and *e*_2_ and which acts as an annotation. Please note that sometimes in the paper we would like to only refer to *e*_1_ and *e*_2_ belonging to the above sets; in this case we use the term pairs.

We detail now the strategy used for computing the different candidate mappings.

### Lexical similarity computing

**Definition 8 (cosine-similarity** Cos_sim_**):** given two *virtual documents*, either *dVD* or *eVD*, representing a first entity as a query *q* and an indexed entity *e*, the cosine similarity between their weighted vectors is:
4

*V*(*q*) *V*(*e*) is the dot product of the weighted vectors and *|V(q)|* and *|V(e)|* are their Euclidean norms. From the above classical cosine similarity formula, the Lucene API introduces some normalisation and boosting factors for the purpose of taking into account the following factors: i) some query terms are more important than others (*queryBoost(q)*); ii) some *documents* (in our case entities) may be more important than others (*docBoost(e)*); iii) for a query with a multiple terms, users can further reward entities matching more query terms through a coordination factor (*coordFactor(q, e)*); and finally iv) in order to avoid known bias introduced by the difference of *documents* length in the classical VSM, Lucene introduces a length normalisation factor (in our case *docLenNorm(e)*) which replaces the Euclidian norms of *V(e)* in formula [4]. Therefore the adapted Lucene score between two *documents* (or entities) *q,* and *e,* known as the Lucene Conceptual Scoring Function (LCSF) is: . *Document* length norm *docLenNorm(e)* and *document* boost *docBoost(e)* are known at indexing time and computed by Lucene in a single value *norm(e*). As each *document* may have several fields (*t*), the single computed value is rather *norm(t, e)*. From the LCSF we define ***Sim***_***lucene***_ (*q, e*) as [44]:
5

where

- *tf*_*t*,*e*_ correlates to the term’s frequency, defined as the number of times term *t* appears in the currently scored entity *e*. ; Where frequency(t) denotes the number of occurrences of *t* within the entity *e*.
stands for inverse *document* frequency. This value correlates to the inverse of *EntityFreq(t)* within the index (the number of entities in which the term *t* appears);
- *coord*(*q*, *e*) is a score factor based on how many of the query terms *q* are found in the specified concept *e*;
- *queryNorm*(*q*) is a normalising factor used to make scores between queries (or even different indexes) comparable;
- *boost*(*t*) is a search time boost of term *t* in the query *q* as specified in the query text;
- *norm*(*t*, *e*) encapsulates a few (indexing time) boost and length factors such as concept boost and field boost.

All the above built-in functions are detailed in the description of the *TFIDFSimilarity* class of the Lucene API available on the library web site^a^ and documentation [44].

Now, let’s assume that ISub(s_1_, s_2_) is the ISub string similarity between two input strings, a measure adapted for ontology matching [50]. Q-Gram (s_1_, s_2_) is the n-gram similarity distance between two texts string, which is simply the number of common/distinct n-grams between two strings [51]. Finally Lev(s_1_, s_2_) is the Levenshtein distance between two strings, which is the minimum number of single-character edits (insertion, deletion, substitution) required to change one word into another [52]. We introduce in the following the lexical similarity.

**Definition 9 (lexical similarity between entities):** given two entities (concepts or properties) *e*_*1*_ and *e*_*2*_ such that *e*_*1*_*∈ O*_*1*_ and *e*_*2*_*∈ O*_*2*_, the lexical similarity *Sim*_*lex*_*(e*_*1*_*, e*_*2*_*)* is defined as*:*6

where ***α, β***, ***γ*** ∈ [0, 1] are respectively the weight for the ISub, Q-Gram and Levenshtein distances (***α*** + ***β*** + ***γ*** = 1).

**Figure 3:**
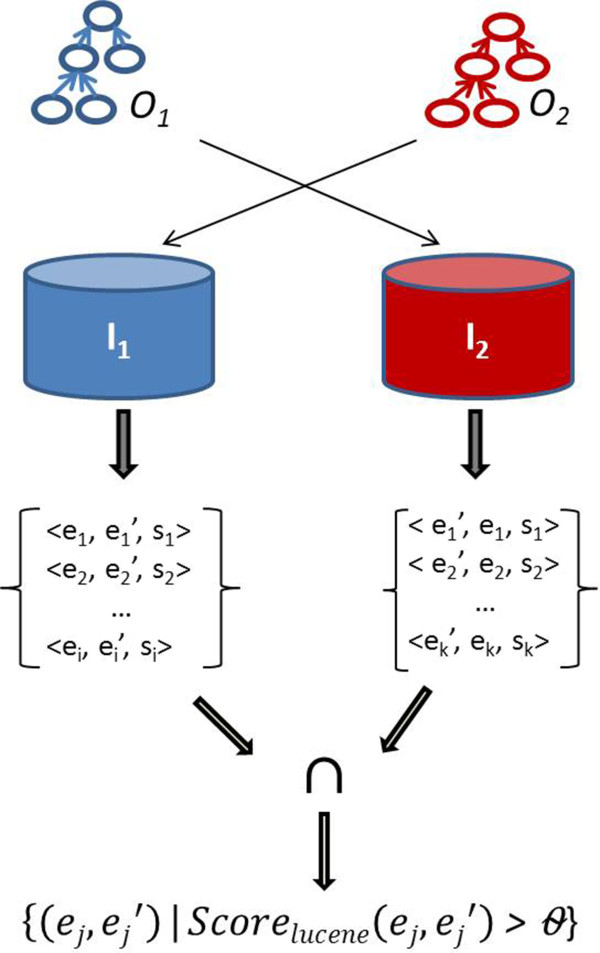
**Lexical similarity computing.**

The objective of the lexical similarity computing is to build the exact candidate sets *M*_*exact*_ and *M*_*prop*_. *M*_*exact*_ is constituted of all triples < *e*_1_, *e*_2_, *Sim*_*lucene*_(*e*_1_, *e*_2_) > such that  and  and *Sim*_*lucene*_(*e*_1_, *e*_2_) is greater than a given threshold. The followed process is depicted in Figure 3.

Given two input ontologies, *O*_*1*_ and *O*_*2*_, and their respective indexes, I_1_ and I_2_, obtained after the indexing step described previously, by using the search component of ServO, we perform an exact search respectively for I_1_ using *O*_*2*_ as search component and for I_2_ using *O*_*1*_. To do so, for each  query from its *direct virtual document* is generated and sent to the index I_2_. Similarly, for each  a query from its *virtual document* is generated and sent to the index I_1_. We intersect the two resulting sets to keep all pairs found from the two way search. From the intersected results, we select the Best-k results (k chosen empirically) that have *Sim*_*lucene*_ greater than a given *MaxScore*. This *MaxScore* is chosen manually. The obtained pairs are filtered out in order to keep only those satisfying a *lexical similarity condition*. This condition is to keep all pairs *< e*_*1*_*, e*_*2*_ *>* such that ***Sim***_***lex***_(***e***_1_, ***e***_2_) ≥θ. To compute *M*_*exact*_, *Sim*_*lucene*_ acts as a pre-filter which compares two entities as a “whole”, regardless of the order of words within a term. For instance, *Sim*_*lex*_ combines finer similarity metrics. In addition, with the use of an index, *Sim*_*lucene*_ allows reduction of the search space.

During the querying process, each *direct virtual document* constituting a query is passed through the same set of filters that are applied during the indexing step.

A similar strategy of computing *M*_*exact*_ is used to compute the similarity between the properties of input ontologies, which generates the *M*_*prop*_ set.

### Extended similarity computing

For extended similarity computing which provides *M*_*extended*_, first the same process as previously described is repeated in order to compute a set from the concepts not yet selected with the exact search. Then, in order to deal with the synonym issue, we implemented a general purpose background knowledge-based strategy. From the set of concepts not selected after the previous phase, we use the WordNet dictionary [53] for retrieval of alternative labels for concepts to be mapped. The idea is to check whether a concept in the first ontology is designed by synonymous terms in the second one. All pairs in this case are retrieved as candidates.

### Contextual similarity computing

The idea of the contextual similarity computing is to retrieve possible new candidate mappings which cannot be found with the terminological description of entities only. Therefore, it introduces the further inclusion of the structure of the input ontologies.

The experiments conducted with our previous approach described in [36] show that the lexical similarity computing provides very highly precise candidate mappings. Therefore, we hypothesise that the *M*_*exact*_ set can be used as a basis for retrieval of new candidate mappings using contextual features.

**Definition 10 (possible context candidates*pcc*):** given two input ontologies, *O*_*1*_ and *O*_*2*_, and a set *M*_*exact*_ of triples obtained by lexical similarity computing, the set of contextual-based candidate pairs, denoted *pcc*, is defined as:
7

**Figure 4:**
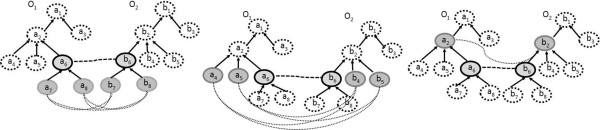
**Strategy for generating possible candidate pairs.**

The strategy of retrieving possible context candidates is illustrated in Figure 4. Let’s assume that (*a*_*6*_, *b*_*6*_) ∈ *M*_*exact*_. The possible contextual-based candidate pairs are then the new candidates from the entourage of (*a*_*6*_, *b*_*6*_).

**Figure 5:**
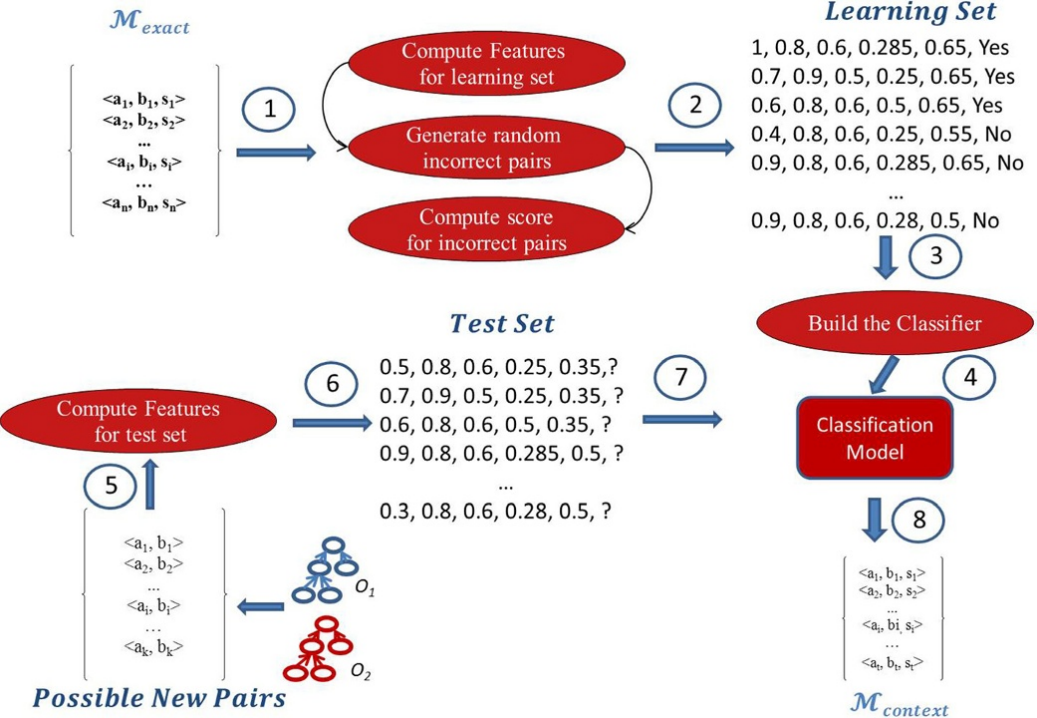
**Machine learning based contextual similarity computing.**

Now we detail how we compute the set *M*_*context*_, which represents the new triples obtained from the contextual similarity computing. We follow a ML strategy to classify the pairs from *pcc* by assuming that the set *M*_*exact*_ is the base learner. Figure 5 gives the followed workflow for the context-based similarity computing. The main idea is to characterise the pairs in *M*_*exact*_ by a set of features and do the same for the *pcc*.

First we generate the learning set from the *M*_*exact*_ set. Each pair in this set, assumed correct, is labelled as “Yes” and we randomly generated incorrect pairs denoted as “No” (see (1) and (2) in Figure 5). To do so, for each pair in *M*_*exact*_ we compute a set of five similarity measures (Q-Gram, Levenshtein, BlockDistance, Jaccard and Monge–Elkam) between the *eVD* (which does not in this case include the properties). Further, we randomly generate a set of incorrect candidate pairs such that for each (*c*_*1*_, *c*_*2*_) ∈ *M*_*exact*_ we obtain . The main idea here is that a concept *c*_*1*_ cannot be mapped both by a concept *c*_*2*_ and its descendant or ancestor concepts. We have chosen five different similarity metrics to cope with short and long text strings. The Jaccard measure [54] computes the number of words two strings have in common divided by the total number of unique words. The Monge–Elkam measure [55] is a simple but effective method of measuring similarity between two text strings containing several tokens, using an internal similarity function, *sim(a, b)*, able to measure the similarity between two individual tokens. The block distance between two vectors *a* and *b* is  where *n* is the dimension of the vectors.

After generating these features, the next step is to build the classifier from the data generated previously ((3) and (4)). We use a DT [56], which was proven to be efficient, and the J48 algorithm implemented within the Weka framework^b^. From the *pcc* we keep as test set all pairs having the computed score *s* = *getScoreSub() + getScoreSup() + getScoreSib()* > φ (φ is chosen manually) and we generate the same features based on the five similarity measures ((5) and (6) in Figure 5). The functions *getScoreSub()*, *getScoreSup()*, *getScoreSib()* compute, respectively, for each possible pair (*c*_*1*_, *c*_*2*_) a score from the sub-concepts pairs, super-concepts and siblings pairs. The idea is to compute the similarity between two concepts *c*_*1*_ and *c*_*2*_ from the similarity between their surrounding concepts, taking into account the depth of these surrounding concepts from *c*_*1*_ and *c*_*2*_. For instance, for the *getScoreSub()*, the sub-concepts of *c*_*1*_ and those of *c*_*2*_ are considered. The sub-concepts that are far apart in terms of depth contribute less to those that are closer.

Finally the set of contextual-based candidate mappings *M*_*context*_ is generated from the test set which is classified using the previously built classification model ((8) in Figure 5).

### Post-processing phase

This step involves enriching the set of candidates mapping, the selection of the best candidates, performing a logical consistency check and finally, if a reference alignment is available, performing the system performance evaluation. The enrichment consists mainly of incorporating those identified, not originally mapped pairs and mapping all of their sub-concepts after the similarity computing phase. The selection of the final candidates from the set *M*_*candidate*_ is performed using a new, improved filtering algorithm from the previous ServOMap implementation [36]. The filtering algorithm implements a greedy selection strategy to select the best candidates based on their scores.

**Figure 6:**
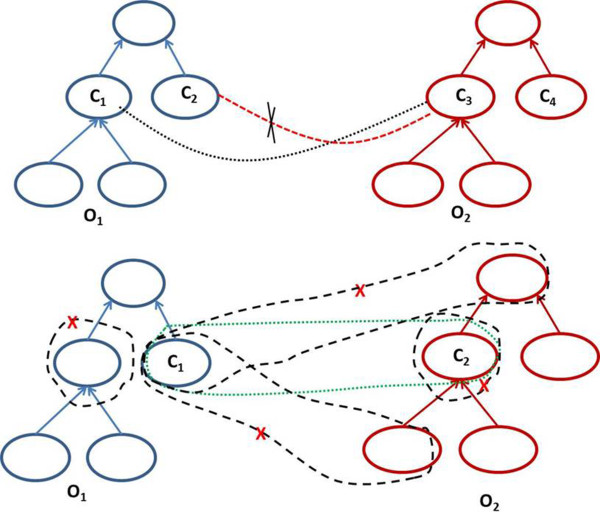
**Discarding incorrect candidate mappings.** The green dashed line of the part (b) of the figure identifies the pair from M_exact_. The dashed black lines with red cross identify the candidate mappings to remove.

The logical consistency check consists of two steps. First we filter out possible incorrect mappings mainly due to the extended and contextual similarity computing which generates less precise candidate mappings than lexical similarity computing. Therefore, two kinds of consistency checks are performed as indicated in Figure 6. The first check (Figure 6(a)) is to discard candidate pairs constituted by disjoint entities if *c*_*1*_ ∩ *c*_*2*_ = ∅ and (*c*_*1*_, *c*_*3*_) ∈ *M*_*exact*_ then we remove any (*c*_*2*_, *c*_*3*_) from the candidate mappings set *M*_*exact*_ . The second check involves removing all *criss-cross* candidate mappings. The idea is to select the best candidates that are not in conflict with the candidate mappings belonging to *M*_*exact*_. In Figure 6(b), if the pair (*c*_*1*_, *c*_*2*_) ∈ *M*_*exact*_ then we discard all generated candidates between *c*_*1*_ and , *c*_*1*_ and  and finally  and *c*_*2*_.

In addition to these *trivial* checks, we reuse the LogMap-Repair facility system [8] to perform logical inconsistency checks. This repair facility has proven to be effective in providing almost clean mappings from the results provided by the ServOMap system [57].

Finally, we have implemented an evaluator to compute the usual precision (*P*), recall (*R*) and F-measure (the harmonic mean) for the generated final mappings if a reference alignment is provided. If *CM* is a set of correct mappings (the reference mappings), and *RM* the set of mappings returned by ServOMap, then these metrics are computed with the following formulas:
8910

## Evaluation and results

In this section we report the evaluation performed for ServOMap and the results obtained. We will first describe the dataset used for the evaluation and then present the different results.

### The dataset used

The dataset used is the LargeBiomed dataset of the OAEI campaign. The LargeBiomed dataset is one of the official datasets which has been provided since 2012 within the context of the OAEI campaign. It is currently the most challenging task in terms of scalability and complexity. It is dedicated to the evaluation of automated large scale matching systems. The ontologies in this dataset are semantically rich and contain tens of thousands of entities. The track consists of finding alignments between the FMA containing 78,989 concepts [31], the SNOMED-CT containing 306,591 concepts [7] and the NCI Thesaurus (NCI) containing 66,724 concepts [32].

For this evaluation, the 2009AA version of the UMLS Metathesaurus is used as the gold standard for the track reference alignments [34]. The Metathesaurus is a very large, multi-purpose, and multi-lingual vocabulary database that contains information about biomedical and health related concepts, their various names and the relationships among them. It is built from the electronic versions of more than 160 biomedical resources including thesauri, classifications, code sets and lists of controlled terms. It is worth noting that as UMLS may contain some incoherencies and is not complete, the performance of an automated matching system could be affected when using a reference alignment from this resource.

In order to measure the behaviour of the matching system according to the size of the input ontologies three matching problems are identified: the FMA-NCI matching problem, the FMA-SNOMED matching problem, and the SNOMED-NCI matching problem as indicated in Table 2, with each problem divided into two subtasks: small and large. According to this table, we have considered six subtasks according to the size of the fragments of the input ontologies. Therefore, for the FMA-NCI problem, the small task consists of matching 5% of the FMA (3,696 concepts) and 10% of the NCI (6,488 concepts) while the large task consists of matching the whole ontologies. For the FMA-SNOMED problem, the small task consists of matching 13% of the FMA (10,157 concepts) and 5% of SNOMED (13,412 concepts). The large task consists of the complete FMA and 40% of SNOMED (122,464 concepts). For the SNOMED-NCI problem, the small fragment consists of 17% of SNOMED (51,128 concepts) and 36% of the NCI Thesaurus (23,958 concepts) while the large task consists of the complete NCI Thesaurus and 40% of SNOMED.

**Table 2:** **Size of input ontologies considered for the different matching problems**

Matching problem	Small task		Large task	
**FMA-NCI**	FMA	NCI	FMA	NCI
	5% - 3,696	10% - 6,488	100% - 78,989	100% - 66,724
**FMA-SNOMED**	FMA	SNOMED	FMA	SNOMED
	13% - 10,157	5% - 13,412	100% - 78,989	40% - 122,464
**SNOMED-NCI**	SNOMED	NCI	SNOMED	NCI
	17% - 51,128	36% - 23,958	40% - 122,464	100% - 66,724

Each cell indicates the percentage of the fragment and the corresponding number of concepts.

### Variants of the ServOMap system used for the evaluation

The evaluation of four versions of the system corresponding to different versions is reported in this paper. Each version corresponds to a particular configuration of the system and/or the implementation of some specific strategies. Therefore we consider the following versions of the system.

- **ServOMap-lt**: this version of ServOMap is a light version of the system in the sense that only one of the input ontologies (the larger one) is indexed during the indexing phase. In this case, entities from the not indexed input ontology are used as queries to search within the built index. It uses the direct description of entities and stemming of labels. The properties and contextual similarity are not taken into account during the matching process; only mappings between concepts are computed. In order to choose the best mapping candidates, Levenshtein distance is used toselect those candidates with the highest similarity measure between the IDs of the concepts. In addition, ServOMap-lt provides *1:n* candidate mappings^c^. That is, a concept from the first input ontology can be mapped to several concepts of the second ontology.
- **ServOMap_2012**: this version indexes the two input ontologies for the retrieval of candidate mappings with high precision. It takes into account both concepts and properties and provides only *1:1* mappings. Both ServOMap-lt and ServOMap_2012 use the built-in cosine similarity implemented within the Lucene API as similarity measure between entities and no any external background knowledge is used.
- **ServOMap_2013**: this version too indexes both input ontologies. It provides *1:n* mappings, meaning in this case that one entity in the first ontology can be matched to several entities in the second ontology and vice-versa. WordNet is used as general purpose background knowledge and thresholds are used to select the candidate mappings during lexical similarity and contextual similarity, as described previously. In addition, the logical consistency repair facility is used during the post-processing phase.
- **ServOMap_V4:** this version is the latest version of the ServOMap system. One of the main differences, compared with ServOMap_2013, is the version of the Lucene API used (a more recent version is used here) and it does not use the LogMap-Repair facility.

From a technical point of view, ServOMap is fully implemented in JAVA as well as the ServO OR on which it relies. The JENA framework is used for processing ontologies in ServO for ServOMap_2012 and ServOMap-lt, and the OWLAPI for ServOMap_2013 and ServOMap_V4.

All the above versions provide only equivalence mappings as ServOMap is currently not able to perform oriented mappings. ServOMap-lt and ServOMap_2012 were included in the OAEI 2012 campaign while ServOMap_2013 was used during the OAEI 2013 campaign.

The SEALS platform [58] is used for the automated evaluation of the first three versions in the context of the OAEI campaign. The SEALS project is dedicated to the evaluation of Semantic Web technologies. It created a platform^d^ to ease this evaluation, organising evaluation campaigns and building the community of tool providers and tool users around this evaluation activity. The overall process of the OAEI campaign using this platform is described on the campaign website^e^.

## Results

The evaluation reported in this section for the three first versions of ServOMap was performed in a server with 16 CPUs allocating 15 GB RAM in the context of the OAEI campaign. The latest version was evaluated using a laptop (Intel Core CPU 2.8 GHz) running under Ubuntu Linux with 6 GB RAM. The computation times are expressed in seconds. The precision, recall and F-measure were computed according to the formulas described in Related work.

We summarise, in the following section, the performance of the different versions on the dataset described above.

### Results of ServOMap_2012

The performance achieved by ServOMap_2012 is summarised in Table 3. For the FMA-NCI matching problem, this system obtained, for the small task, an F-measure of 85.5% with a precision of 99% by providing 2,300 mappings. For the large task we observed a small decrease of the performance with an F-measure of 82.8%.

**Table 3:** **Performance achieved by the ServOMap_2012 version on the LargeBio dataset**

***ServOMap_2012***	Task	#Mappings	Precision	Recall	F-Measure	Time
**FMA-NCI**	Small	2,300	99%	75.3%	85.5%	25
	Large	2.413	93.3%	74.4%	82.8%	98
**FMA-SNOMED**	Small	6,009	98.5%	65.7%	78.8%	46
	Large	6,272	94.1%	65.5%	77.3%	315
**SNOMED- NCI**	Small	10,829	97.2%	55.9%	70.9%	153
	Large	12,462	83.5%	55.2%	66.4%	654

The FMA-SNOMED matching problem took longer to process than the FMA-NCI one. There were more entities to compare within the input ontologies of this task. For the small task of this matching problem, ServoMap_2012 obtained an F-measure of 78.8% by providing 6,009 mappings while for the large task it achieved an F-measure of 77.3%, a small decrease compared to the small task.

The SNOMED-NCI matching problem presented more entities to compare between the input ontologies. The observed computation time was thus greater than with the previous matching problems. ServOMap succeeded in providing 12,462 mappings for the large ontologies with an F-measure of 66.4% for the SNOMED-NCI matching problem.

Regarding the computation times, the fastest task was, unsurprisingly, the small task of the FMA-NCI matching problem, which consisted of a relatively very small portion of entities. The system performed this task in 25 seconds while the large SNOMED-NCI task took 11 minutes.

### Results of ServOMap_lt

Table 4 summarises the results achieved by ServOMap-lt. For the FMA-NCI matching problem, the system provided 2,468 mappings with an F-measure of 88.8% and the greatest precision while for the large task we observed the same behaviour as in the ServOMap_2012 case: a slightly decrease of the performance with an F-measure of 85.2%. For the FMA-SNOMED matching problem, ServOMap-lt provided 6,563 mappings for the small task against 6,563 for the large task, which corresponds respectively to 81.4% and 79.7% of F-measure. Similar to the previous system, the SNOMED-NCI matching problem was more difficult to handle. The F-measure was respectively 73.7% and 67.8% for the small and the large task with a relatively stable recall of around 59%.

**Table 4:** **Performance achieved by the ServOMap_lt version on the LargeBio dataset**

***ServOMap-lt***	Task	#Mappings	Precision	Recall	F-Measure	Time
**FMA-NCI**	Small	2,468	98.8%	80.6%	88.8%	20
	Large	2,640	91.4%	79.8%	85.2%	95
**FMA-SNOMED**	Small	6,348	98.5%	69.4%	81,4%	39
	Large	6,563	94,5%	68,9%	79,7%	234
**SNOMED-NCI**	Small	11,730	96%	59.8%	73.7%	147
	Large	13,964	79.6%	59%	67.8%	738

We can notice here that ServOMap-lt performed better than ServOMap_2012 in regard to the F-measure and the computation times (expect for the large SNOMED-NCI task). We discuss this behaviour in Evaluation and results.

### Results of ServOMap_2013

The results of ServOMap_2013 are described in Table 5. Compared to the previous systems, the FMA-NCI was the easiest task to perform. The system obtained an F-measure of 87.7% for the small task (2,512 mappings) against 76.3% for the large one (3,235 mappings). We observed a drop of about 0.10 points between the two subtasks. For the FMA-SNOMED matching problem, the decrease of the F-measure was less significant: from 81.4% for the small task to 79.7% for the large one following the same behaviour as the ServOMap_2012 system for this matching problem. The results for the SNOMED-NCI matching problem were, for the F-measure, respectively, 76.1% and 71.8% for the small and the large task, with a noticeable drop in terms of precision (from 93.3% to 82.2%). It is worth noting that overall, the computation times significantly increased compared to the two previous systems.

**Table 5:** **Performance achieved by the ServOMap_2013 version on the LargeBio dataset**

***ServOMap_2013***	Task	#Mappings	Precision	Recall	F-Measure	Time
**FMA-NCI**	Small	2,512	95.1%	81.5%	87.7%	141
	Large	3,235	72.7%	80.3%	76.3%	2,690
**FMA-SNOMED**	Small	5,828	95.5%	62.2%	75.3%	391
	Large	6,440	86.1%	62%	72.1%	4,059
**SNOMED- NCI**	Small	12,716	93.3%	64.2%	76.1%	1,699
	Large	14,312	82.2%	63.7%	71.8%	6,320

### Results of ServOMap_V4

Table 6 describes the results achieved by the ServOMap_V4 system. For the FMA-NCI matching problem, the system obtained 89.4% (2,725 mappings) and 79.3% (3,163 mappings) respectively for the small and the large task. For the FMA-SNOMED task there was a significant increase in terms of recall compared to the previous system which led to better F-measures of 83.4% and 78.1% respectively for the small and the large tasks. Regarding the SNOMED-NCI matching task the F-measure was 74.4% for the small task (13,047 mappings) and 68.4% (15,525 mappings) for the large task. We noted that the computation times of ServOMap_V4 and ServOMap_2013 were roughly similar when the repair facility time is not taken into account.

**Table 6:** **Performance achieved by the ServOMap_V4 version on the LargeBio dataset**

***ServOMap_V4***	Task	#Mappings	Precision	Recall	F-Measure
**FMA-NCI**	Small	2,725	94.3%	85%	89.4%
	Large	3,163	71.1%	83,6%	79.3%
**FMA-SNOMED**	Small	6,978	95.5%	74%	83.4%
	Large	7,940	83.3%	73.46%	78.1%
**SNOMED- NCI**	Small	13,047	90.9%	62.9%	74.4%
	Large	15,525	75.7%	62.3%	68.4%

Finally, Table 7 presents the performance achieved by ServOMap_V4 on the small fragment of the input ontologies of the LargeBiomed dataset when coupled with the LogMap-Repair logical consistency facility check. As can be seen, for most of the cases, the precision increased slightly while the recall decreased. Overall, compared to the ServOMap_V4 system alone, the F-measure is lower when the repair facility is used. One of the factors which caused this could have affected the step where the repair facility is used, which could have been either at the end of the matching process or after the lexical computing similarity.

**Table 7:** **Use of the LogMap repair facility with ServOMap_V4 on the small fragment of the input ontologies**

	#Mappings	Precision	Recall	F-Measure
**FMA-NCI**	2,651	95.2%	83.5%	88.9%
**FMA-SNOMED**	6,402	95.4%	67.9%	79.2%
**SNOMED- NCI**	12,587	92.7%	61.9%	74.2%

## Discussion

The ontology matching field is maturing. We have noticed significant progress of the systems included in the 2012 and 2013 edition of OAEI. However, dealing with large ontologies still remains a key challenge. The ServOMap system, an automated a generic ontology matching system, has proven to be efficient when dealing with large scale matching tasks. It is based on IR techniques which combine the use of the lexical description of entities to be matched and their contextual information.

Our findings suggest that an IR-based approach, relying on the terminological description of entities, combined with a structural similarity approach is very effective for the matching of large ontologies. They also show that it is possible to compute mappings with very high precision by using lexical similarity computing and dealing with the complexity of matching large ontologies without using blocking strategy, in contrast to the approaches described in Related work.

Regarding the participating systems or configurations in the OAEI campaign, 15 out of 23 and 13 out of 21 were able to cope respectively with at least one of the tasks of the LargeBiomed track matching problems at OAEI 2012 and 2013 [59, 60]. Our system was among the best system in terms of F-measure for all the variants and in terms of computation times for ServOMap_2012 and ServOMap-lt. ServOMap_2012 was able to provide mappings with the best precision for the task of matching the FMA, NCI and SNOMED. As shown in the results described previously, the computation times increased drastically for ServOMap_2013 and ServOMap_V4. Two factors contributed to this. First, in these latter versions, we assume that the F-measure is a more important factor than the computation time as in several use case scenarios, mappings could be computed in a batch mode and provided then to the running system. Second, the introduction of several string similarity metrics, computed for each candidate pair, as well as the new contextual similarity strategy based on ML, impacted the computation times of ServOMap.

Regarding the behaviour of ServOMap_2012 and ServOMap-lt, the performance of the latter was better in terms of F-measure for all the tasks described above. This could be explained by the fact that the recall of ServOMap-lt is a step ahead due to its ability to compare the labels of the concepts simply in the different tasks. Using the *tf.idf* measure, completed by a Levenshtein distance-based selection of best candidates, could be sufficient for the kind of resources within the LargeBiomed dataset. This finding is in line with the results obtained by [61] after comparing different string similarity metrics of ontology matching. ServOMap_2012 has proven to be more stable and efficient for the other tracks of OAEI [59], in particular for relatively small ontologies associated with poor terminologies. The use of the intersection of results provided by the search over the two indexes built from the input ontologies makes the ServOMap_2012 system too restrictive, to the detriment of the recall, but provides highly precise mappings. In addition, for the computation times, ServOMap_2012, in contrast to ServOMap_lt, performs the indexing of both input ontologies, which could be time consuming. However, because of the fact that ServOMap-lt uses the Levenshtein distance in addition to the indexing and searching step to select the best candidates result, the computation times for the large SNOMED-NCI matching problem are greater when using ServOMap_2012 (more than 13,000 returned mappings for ServOMap-lt) (Table 4). This matching problem has more entities to compare.

The use of the WordNet general purpose background knowledge for the newer version, as well as the new ML-based contextual similarity, had a positive impact on the performance of the system in terms of recall improvement. This is particularly true for the SNOMED-NCI matching problem where ServOMap_2013 gained 4.4% over ServOMap_lt and 8.3% over ServOMap_2012 without decreasing the precision.

Regarding the use of different string similarity metrics for the computation of the features of the pairs used in the ML-based contextual similarity computing, these were selected to optimise results obtained for both short and long text string comparisons. In the present study, we did not change these measures to analyse the impact on the performance of the system. Such an evaluation will be conducted in a future study. For the choice of these metrics, we can benefit from the results of the evaluation conducted by [61].

Regarding efficient handling of scalability, while other similar systems, when dealing with large ontologies, rely mainly on blocking techniques to reduce the search space [29, 30, 33], ServOMap relies only on IR techniques to build indexes from input ontologies used for similarity computing.

We discuss in the following some aspects of the strategy used and the performance of the ServOMap system and highlighting similar research works.

### Combining lexical and contextual strategies in ontology matching

Lexical and contextual or structural similarity computing have been used in several approaches of automated ontology matching at large scale in the biomedical and life sciences domain [62–65]. In this domain, the UMLS is widely used as a resource. A combination of lexical and semantic approaches is used in [63] to generate mappings between SNOMED-CT and the ICD-9 thanks to the use of the UMLS as knowledge base. The semantic approach makes use of semantic relationships between UMLS concepts to find mappings, while the lexical mapping uses MetaMap, a tool used to recognise UMLS concepts in texts. The combined approach achieved a precision of 27% and a recall of 43%. In the current version of ServOMap, the UMLS is not used as input resource, only as a resource which provides the reference alignments for evaluation of the system. However, it could be interesting to use some of the components of the UMLS in the future, in particular the semantic group, in order to check the validity of provided mappings. In [64] an automated approach to mapping the EMTREE thesaurus to the complete UMLS Metathesaurus is described. The approach uses the web service *NormalizeString* provided by the UMLS Knowledge Source Server to identify similar strings across the input terminologies in the lexical step. Then, the generated candidates are validated using a structural strategy which consists of computing paths to top-level concepts and checking compatibility across the external terminology and the UMLS Metathesaurus. A global precision of 78% is obtained by this approach. However, the only available evaluation is between the EMTREE, which is not freely available, and the UMLS. Therefore, it is not possible to check whether the performance is similar for other resources. In contrast, ServOMap is a generic approach which has been evaluated using standard benchmarks provided by the OAEI campaign, with various datasets.

Zhou and colleagues used Natural Language Processing techniques to map a drug dictionary to RxNorm [65]. They mapped about 6,000 terms from Partners Master Drug Dictionary and 99 of the top prescribed medications to RxNorm. The mapping was performed at two levels: term level (by performing string-based matching using specific pre-processing techniques) [66] and concept level (relying on routes group). The evaluation showed an F-measure of 84.2% for the concept level mapping. For the closest task in terms of scalability, our system achieved a performance ranging from 85.5% and 89% for the small task of the FMA-NCI consisting of matching 3,696 concepts of the FMA and 6,488 of the NCI.

### The use of the Lucene IR library

The use of the Lucene IR library in ontology matching is not new. The YAM++ system uses it in its IR-based strategy, in particular to index a larger sized ontology. For large scale ontology matching YAM++ has recently introduced the Lucene ranking score [67] as a metric similarly to that used in the ServOMap system. This system was among the top three systems along with ServOMap during OAEI 2012 and obtained the overall best F-measure as well in 2013. YAM++ indexes only the description of the entities of the larger sized size ontology. This strategy of indexing one of the ontologies is similar to the one used in ServOMap-lt.

Pirro and Talia introduced the LOM (Linguistic Ontology Matcher), a linguistic approach based on IR techniques using the Lucene library [68]. It gathers different kinds of linguistic information for the entities of the source ontology into a Lucene index. Mappings are then obtained by exploiting values of the entities of the target ontology as search arguments against the index created from the source ontology. The Protégé API^f^ is used to process the input ontologies. Similar to the previous case, the LOM approach is very close to the strategy implemented in ServOMap_lt as only one of the input ontologies is indexed. However it differs in the sense that LOM uses the same set of predefined features for each entity while in ServOMap they are dynamically generated.

### Effect of logical assessment on mapping repair

According to Jiménez-Ruiz *et al.* [57] the application of mapping repair techniques has a significant impact on the quality of the mappings with respect to their logical coherence. They conducted an empirical evaluation using two state-of-the-art mapping repair systems, Alcomo [69] and LogMap-Repair [57]. The evaluation was conducted using the results provided by the best systems from the OAEI 2012 LargeBiomed track, including ServOMap. We then experimented by reusing the LogMap-Repair logical consistency repair facility in ServOMap_2013 and on the results provided by ServOMap_V4 with the small task of the LargeBiomed dataset. Even though our goal was to improve the performance of ServOMap, we noticed that in some cases the logical assessment is too aggressive as it discards some correct candidate mappings and negatively impacts the F-measure, while reducing the number of incoherence mappings. This is in line with Pesquita *et al.* [70] who reported recently that the repair technique employed for the LargeBiomed track to create a reference alignment removes a considerable portion of correct mappings which affects the performance of the evaluated systems. We have to further investigate this issue in order to identify the best strategy for use of the repair facility.

### Limitations of the ServOMap system

ServOMap is well adapted for life sciences ontologies because these ontologies used to rely on rich terminological description including several synonym terms for each entity, which is suitable for IR techniques. But, the strategy followed in our approach is heavily based on lexical similarity computing. Indeed, its results are used as input for the contextual similarity computing. This is a major limitation because when dealing with ontologies with poor lexical descriptions, the system may provide results with low recall.

Further, currently the different thresholds used in the system are chosen manually, which leads to the use of the same filtering value regardless of the matching task. It would be interesting to dynamically choose these thresholds according to the matching case and the parameters computed during the metrics computation step.

In addition, ServOMap is able to provide only equivalence mappings, which is a drawback when dealing with some matching tasks as the recall could be negatively affected if the reference alignment is comprised of subsumption and disjointness relationships.

## Future work

According to the achieved performance and the limitation raised above, there is room for improvement in the ServOMap system to address the challenges of large scale ontology matching [17].

### Interactive matching

**Figure 7:**
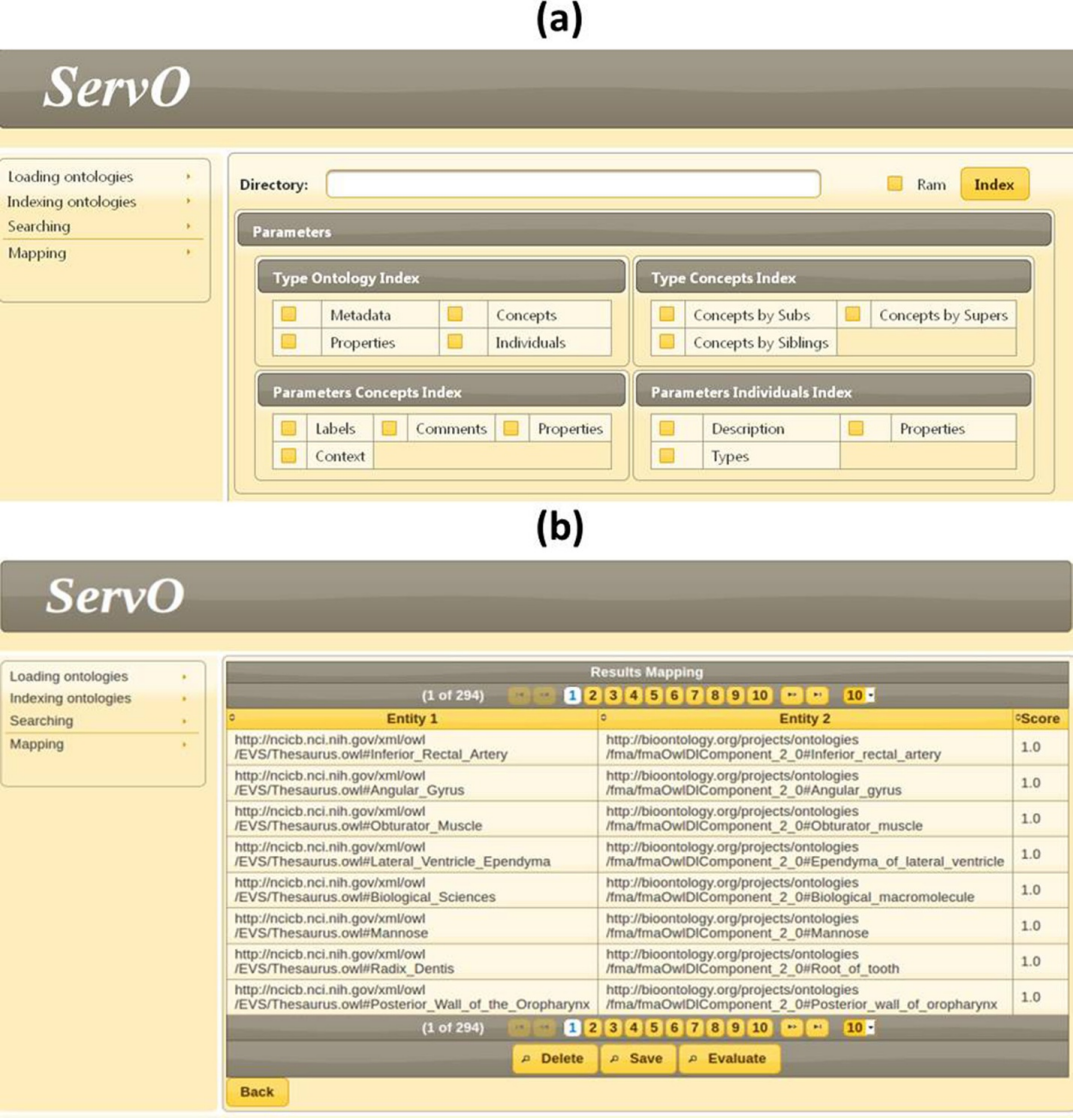
**Graphical user interface of the system: parameters (a) and mappings (b).**

We plan to introduce interactive matching strategy in ServOMap during large scale ontology matching in order to improve the recall in particular. Currently, as can be seen in Figure 7, our system only provides a user interface to set up the different parameters of the matching process (part (a) of the figure) before the automated generation of mappings (part (b) of the figure). However, automated generation of mappings can be seen as the first step in an ontology matching process [12]. Indeed, taking into account the user involvement can improve the quality of provided mappings. The OAEI campaign has introduced, since the 2013 edition, a new track dedicated to interactive matching. Only four systems among those utilised addressed this challenge [60]. The results showed that LogMap and the AgreementMakerLight framework had improved their recall thanks to the introduction of this strategy [60]. The approach proposed by [28] constitutes an interesting direction to investigate.

We also plan, along the lines of interactivity, to improve the currently available user interface. The objective is to take into account the possible evolution regarding, in particular, the user involvement and interactive matching strategy.

### Oriented and cross-lingual mappings

The current version does not take into account the matching of two input ontologies described in two different languages. For instance, comparing an ontology with terms in English to an ontology with terms in German. Therefore, we plan to investigate an approach for cross-lingual ontology matching. In addition, ServOMap can only provide equivalence mappings. The idea is to complete our matching strategy by providing users the ability to compute subsumption or disjoint relationships between entities of two input ontologies. Regarding these oriented mappings, at a large scale, an important challenge will be the evaluation of the provided mappings because of the availability of suitable reference alignments.

Finally, it is worth conducting an extensive evaluation of the impact of the different parameters, the type of matching problems and the characteristics of the input ontologies on the performance of the ServOMap system. Moreover, we intend to further investigate the logical assessment of computed mappings [71], which could help improve the quality of the mappings that are provided.

## Conclusion

We have presented in this paper the ServOMap large scale ontology matching system. ServOMap is a proposed generic approach combining lexical and contextual strategies for the retrieval of candidate mappings. Thanks to the use of an IR-based indexing strategy, the system can efficiently cope with large ontologies.

We have described the results achieved by the system using a standard benchmark with matching problems provided by the OAEI LargeBiomed track. The results for this track showed that ServOMap was among the top featured systems. They also showed that the recent introduction of a general purpose background knowledge and ML-based strategy for contextual similarity computing has a positive impact of the F-measure while increasing the computation times.

## Endnotes

^a^https://lucene.apache.org/.

^b^http://www.cs.waikato.ac.nz/ml/weka/.

^c^In some situations, like for the reference alignment provided for the OAEI Library reference, several equivalence candidate mappings are correct with an 1:n. We take into account all the selected mappings for evaluating the performance of the system. The evaluation made for the versions which provide 1:n candidate mappings shows an increase of the performance.

^d^http://www.seals-project.eu/.

^e^http://oaei.ontologymatching.org/2012/seals-eval.html.

^f^http://protege.stanford.edu/.

## Abbreviations

ADR: Adverse drug reactions
DT: Decision tree
FMA: Foundational model of anatomy
GOMMA: Generic ontology matching and mapping management
ICD: International classification of diseases
IR: Information retrieval
KOS: Knowledge Organisation Systems
LCSF: Lucene conceptual scoring function
LOM: Linguistic ontology matcher
ML: Machine learning
NCI: National Cancer Institute
OAEI: Ontology alignment evaluation initiative
OR: Ontology repository
SNOMED-CT: Systematized Nomenclature of Medicine- -Clinical Terms
SVM: Support Vector Machine
UMLS: Unified Medical Language System
VSM: Vectorial space model.
